# Ten simple rules for implementing a successful field season

**DOI:** 10.1371/journal.pcbi.1012189

**Published:** 2024-06-20

**Authors:** Lindsey R. Perry, Rebecca L. Kelble, Valerie N. Brewer, Cara E. Christensen, Mark E. Kerstens, Terrah M. Owens, Megan A. Sampognaro, Dorothy L. Zahor, Rachel A. Zitomer, Suzanne H. Austin, Jamie M. Cornelius, Jonathan B. Dinkins, Sarah J. K. Frey, Cecelia E. Frisinger, Stephanie M. LeQuier, Carl G. Lundblad, Jamie Oskowski, Hallie R. Perlman, William J. Price, Richard Rich, Kayla A. Ruth, Vanessa M. Schroeder, Shawn B. Szabo, James W. Rivers

**Affiliations:** 1 Department of Animal and Rangeland Sciences, Oregon State University, Corvallis, Oregon, United States of America; 2 Department of Fisheries, Wildlife, and Conservation Sciences, Oregon State University, Corvallis, Oregon, United States of America; 3 Department of Integrative Biology, Oregon State University, Corvallis, Oregon, United States of America; 4 Department of Forest Engineering, Resources, and Management, Oregon State University, Corvallis, Oregon, United States of America; 5 Department of Forest Ecosystems & Society, Oregon State University, Corvallis, Oregon, United States of America; 6 Department of Animal and Rangeland Sciences, Oregon State University and Eastern Oregon Agricultural Research Center-Burns, Burns, Oregon, United States of America; Carnegie Mellon University, UNITED STATES

## Introduction

Science frequently requires the collection of data in a field setting that is markedly different from the stereotypical laboratory often associated with scientists. For example, archeologists may collect data from field excavations to address questions about the foundations and development of civilization, and epidemiologists often conduct research under field conditions to learn about disease pathology in response to outbreaks. Successful field data collection requires considerable planning and organization to navigate challenges that stem from conducting research in such uncontrolled settings. Inadequate preparation can limit data quantity and quality, and may even create safety risks. Thus, a concerted effort that starts well before data collection begins and extends throughout the field season is critical to project success.

The skills required to implement successful field work often develop with time and experience. However, we note the need for a guiding framework to accelerate and facilitate this process among new researchers, including graduate students who may be inexperienced or are constrained by time. Here, we draw upon our collective experience conducting field research in the natural sciences—covering >200 cumulative field seasons undertaken in 12 countries and 29 US states—to develop rules that we have found to be essential for implementing a successful field season. Our goal is to provide researchers in the natural sciences who have limited field experience with a suite of considerations that we have found to be useful when planning a field season. Of note, many of our rules are germane to field data collection efforts in other disciplines, especially those conducted by a team in the field. Indeed, those of us who supervise graduate students often share these rules with students who are planning their first field season.

In assembling these rules, we have made several assumptions about our intended audience, whom we refer to as “lead researchers”: (1) they can draw upon their prior field experience from a previous position(s) (e.g., as a field technician); (2) they have already identified research questions and selected locations for undertaking fieldwork; (3) their study design and data collection methods are feasible and scientifically sound; and (4) their research is being undertaken with consideration of the safety and well-being of field personnel and any organism(s) they are studying. Importantly, the rules we have assembled are not focused on the nuances of field sampling but instead address the broader organizational and logistical challenges that come with implementing a successful field season. Our rules are not meant to be an exhaustive list, and the specifics of a particular project may require additional planning not discussed here. For example, conducting international field research requires considerations that are beyond the scope of this paper. Nevertheless, we regard these rules as particularly useful for guiding lead researchers that lack experience in planning and preparing for the challenges—and the rewards—of field research.

## Rule 1: Start planning early

A successful field season should begin early to allow enough time to complete the many disparate tasks that fall under the project planning umbrella. Before data collection, the lead researcher must develop concise and coherent scientific protocols, secure relevant permits, obtain necessary materials and equipment, and hire and train a competent field crew, among other tasks. This workload necessitates that project planning starts months ahead of time. In our experience, 5 to 6 months is often the minimum time required to ensure that all tasks are completed on schedule [1]. Although obvious to many, we emphasize that lead researchers should start by developing a list of project-specific tasks and estimate their expected time to completion, and then prioritize those that take the longest to complete or are inflexible with respect to when they must be completed. For example, obtaining research permits, gaining permission for land access, and securing field accommodations are often lengthy processes. For lead researchers working with vertebrates, permits are often required at multiple administrative levels (e.g., university, state, and federal), and some of these groups (e.g., institutional review boards) may meet only on a periodic basis. In addition, revisions to animal handling and care protocols may be required [2], resulting in project delays if revisions to permit proposals are required.

**Fig 1:**
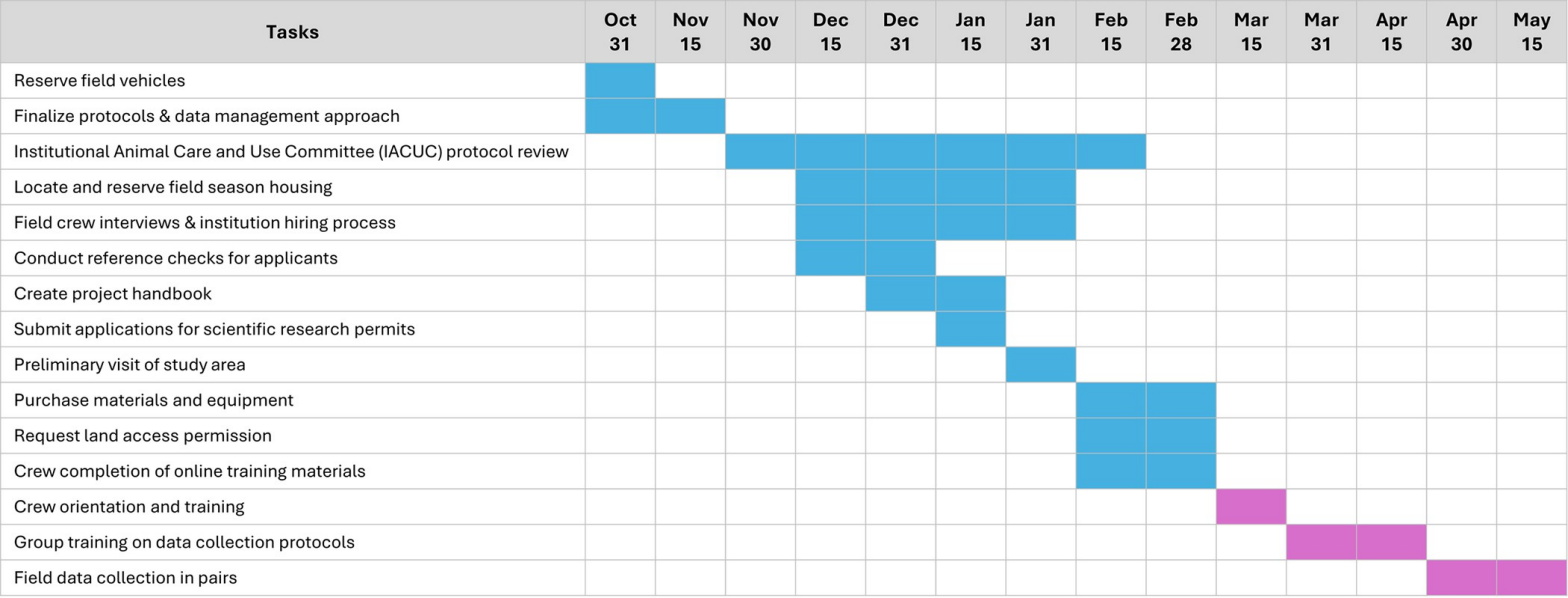
An example of a Gantt chart, a useful project management tool that was created to prepare for a research project that began fieldwork on March 15th. Rows represent distinct tasks and columns represent the time period during which the tasks are planned; color-coding the tasks helps to quickly delineate among tasks that are scheduled before and after the field crew begins work.

Because of the numerous and diverse tasks required for planning fieldwork, we recommend developing a detailed timeline that starts several months prior to the field season and extends through the entirety of when data are collected. One effective project management tool is the Gantt chart (Fig 1), which visually depicts tasks with an associated timeline for completion [3]. Using a visual management approach can be especially useful for planning multiple tasks that must be completed within a similar timeframe and quickly detecting issues related to the timing of events. Lead researchers should schedule time at the beginning of the field season for crew orientation and training, including safety training (see Rules 3 and 5) and informal interactions for crew members to provide a welcoming atmosphere and foster team cohesion [4–7]. Providing extra time to complete tasks early in the season also allows flexibility, troubleshooting, and the ability to change directions which often is needed when implementing a new project (see Rule 9) [8].

## Rule 2: Be thoughtful and deliberate about personnel decisions

In many cases, lead researchers will benefit from assistance from others when collecting field data, and selecting a crew member(s) for the project seems like a simple task. Nevertheless, our experience has found that being thoughtful and deliberate when making personnel decisions results in mutual benefits for both the lead researcher and their crew. To begin, lead researchers should first determine the key attributes that will allow crew members to be successful in their duties; these might include educational background and technical skillsets, as well as individual characteristics such as having close attention to detail, being flexible and open to change, demonstrating self-motivation, following through on tasks, and keeping a positive attitude under challenging conditions [9]. Lead researchers should create a job advertisement that includes a detailed job description, highlights the required and desired skillsets of applicants, provides information about working conditions and compensation, and outlines the nonfinancial benefits an applicant will gain if selected. Using the identified key attributes, lead researchers should prepare interview questions that provide insight into the applicant’s relevant skillsets and prior work experience. Because it may be difficult to evaluate an applicant’s proficiency with some skills through their application materials, we recommend that references should be contacted (see below). For many field projects, techniques can be learned while on the job, so lead researchers should carefully consider which skillsets can be developed during the course of fieldwork and which must be fully developed at the start of a position. Having a mixture of experience levels on a field crew may allow the more experienced crew members to engage in peer-to-peer learning with their less experienced crewmates. This approach provides an opportunity for those crew members who lack field experience to gain valuable skillsets that allow them to advance in their careers, while also providing opportunities for other crew members to gain experience training their peers.

When evaluating potential candidates, it is essential to remain objective and to minimize unconscious bias as much as possible. One approach that we have found helpful is to employ an application process whereby key attributes required for the position are evaluated independently of demographic information or potentially limiting factors. This approach is a small but crucial step that lead researchers can take to diversify their crews, which can ultimately broaden the scope of the crew’s problem-solving capabilities [10] and, more broadly, help to promote diversity in the sciences [11]. When interviewing applicants, lead researchers should be mindful that applicants also use interviews to consider how well a project fits their needs and interests. Therefore, being transparent about what the position entails is essential so that applicants can assess whether a project fits their professional and/or personal objectives. We have found it beneficial to provide a brief summary at the start of an interview that covers many details of the project—such as an overview of the research, work schedule, and compensation—as doing so helps to be efficient with limited time during the interview and gives applicants with the opportunity to ask more nuanced questions regarding the position. Another critical part of the evaluation process that can easily be overlooked is contacting references to assess applicants’ skillsets as they relate to the project, their willingness to learn and grow in a position, and how they handle adversity and change, the latter being hallmarks of field research. Often, the most informed discussions come from phone calls with references instead of written responses, as many references are more candid during a personal conversation. After selections are made, hiring paperwork should be submitted to the requisite human resources department as soon as possible, well before fieldwork begins. Although hiring processes are institution-specific, we have found that it typically takes at least several weeks before a candidate is cleared to work; thus, hiring is another example of making sure to start project planning early (i.e., Rule 1).

## Rule 3: Dedicate adequate time for training

**Fig 2:**
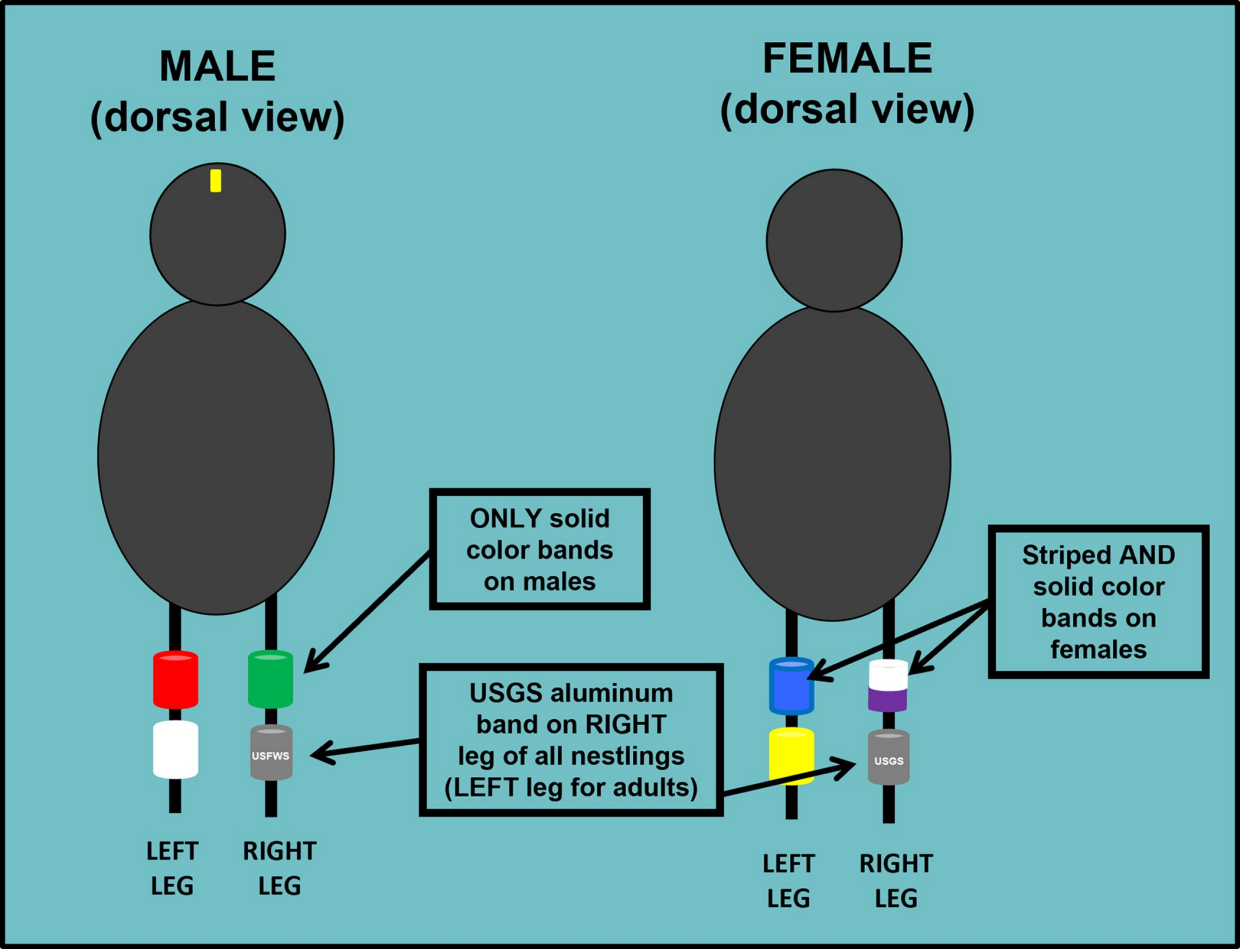
An example of a figure that was used in a project handbook to improve understanding of a detailed protocol for attaching leg bands to nestlings of the Black-backed Woodpecker (*Picoides arcticus*). The study required that unique combinations of metal and colored leg bands to be attached for individual identification after capture, and this diagram was developed to avoid confusion over which leg was being referenced when describing band attachment. In this case, the diagram also served to emphasize the important point that only females should have striped leg bands, which further facilitates identification of sexes under field conditions.

Spending adequate time training field crew members is a key step to a successful field season. In principle, proper training increases employee motivation and confidence, and improves work performance [12], which can translate to higher-quality data collection; it is also crucial for the safety of the field crew (see Rule 5). Lead researchers should review protocols with field crew members well before the start of data collection to ensure field protocols are fully understood, and asking crew members to demonstrate their understanding of protocols is a great way to ensure they are understood. Appreciating how the data will be used and contribute to the field of study also provides important context for crew members to appreciate the full importance of their data collection efforts. Emphasizing that accurate data collection is the goal is also important to ensure data integrity. In addition, adequate time is needed to walk through data collection protocols thoroughly, discuss standard operating procedures (SOPs), and work through other components of a project handbook (see Rule 6). A good approach is to collect “practice” data for each protocol with the field crew prior to the start of actual data collection to clarify any confusion and ensure consistency in data collection among all crew members. Practicing data collection protocols may also bring up questions that can be discussed or explained in a group setting or identify unclear directions or protocol shortcomings that can be corrected prior to the start of actual data collection. Such training also provides an excellent opportunity to introduce specific equipment and troubleshoot potential issues before heading into the field. In addition, we have found that figures accompanying written protocols (e.g., Fig 2) can enhance understanding of data collection procedures, especially for protocols that can be easily misunderstood or are highly detailed. An additional benefit of sufficient training is that a well-informed crew member can serve as an ambassador for a research project to stakeholders and other interested parties that may be encountered.

## Rule 4: Cultivate an inclusive and positive work environment

Fostering a positive work environment is crucial to productivity because it can lead to a more satisfied crew, higher quality data collection, and long-lasting professional relationships [13]. A positive work environment begins with effective communication, starting with the first day of interaction with the crew, which takes place well prior to the start of the field season. Lead researchers should provide clear expectations via a project handbook (see Rule 6) and take the time to ensure that the expectations are understood by crew members. It is also useful to let crew members know that constructive feedback is welcome, which serves to let them know that their voices are heard and welcomed. In our experience, some crew members, through their direct involvement in fieldwork, can provide valuable insights that help to solve logistical issues and make for more efficient data collection.

For the benefit of the whole crew, lead researchers should explain that despite being in a work environment that differs from that of a lab or office, all members of the field crew are held to high professional standards of appropriate behavior. Lead researchers should establish a written code of conduct for their team, including explicit consequences for violations and information on reporting incidents [14,15]. Lead researchers should lead by example by following the code of conduct and using inclusive language. Creating an inclusive environment also requires recognizing that people with different backgrounds may have differing levels of experience with such topics as outdoor sanitation practices and field hygiene. Lead researchers should outline protocols regarding eliminating waste [16] and as well strategies for navigating menstruation while in the field [17]. Additionally, lead researchers should consider providing some of the field equipment necessary for crew members (e.g., backpacks or tents). One of the major barriers that prevent marginalized communities from joining the natural resource field is the financial burden [18] and providing such equipment can promote a more diverse and inclusive work environment.

Interpersonal conflicts among crew members can become a source of tension during the field season and are more likely to occur when crew members are housed together. Each crew member is different regarding what they need to be present and ready for each day in the field; some require time to themselves each day to “recharge,” whereas others may prefer an environment where their interactions with their work colleagues extend well beyond work hours. Given such variation, lead researchers should encourage crew members to express their individual needs at the start of the field season when discussing ways to foster a respectful work environment. In those cases where the field crew is housed together, it is essential to ensure that all crew members are comfortable with sleeping and bathroom arrangements. Lead researchers should explain housing details during the interview so that arrangements are clear well before a position is to be offered. Lead researchers should take into consideration housing preferences (e.g., same gender versus mixed gender) but be forthright with what the specific housing options can realistically accommodate. It can also be valuable to work with the crew to develop a plan for undertaking regular household tasks (e.g., taking out the trash, keeping organized, cleaning shared living spaces) to encourage equal contributions for a positive living environment.

Even when expectations are clearly delineated, issues can and do arise, and lead researchers should anticipate and be prepared to address such issues ahead of time. If intervention is needed, it is essential to keep an open mind, listen to both parties, and solicit input about potential resolutions before making any decisions. Because some issues require intervention from a more senior member of the project team (e.g., principal investigator) or even an outside group (e.g., office of Equal Opportunity and Access), it is essential to understand how conflicts and personnel issues should be handled and when to bring in outside assistance prior to the field season. Lead researchers should consider how communication about conflicts might work in remote situations and what contingency plans might be necessary if issues arise. For transparency, lead researchers should provide contact information for their immediate supervisor or an affiliated ombuds in the event a crew member feels uncomfortable approaching the lead researcher for help with an issue. By being proactive and cultivating a positive work environment, lead researchers will not only develop the trust of crew members but will be better positioned to work through such issues with minimal disruption and lost productivity.

## Rule 5: Prioritize safety from day one

Prioritizing safety begins well before the start of the field season and continues until the end of the project [19,20]. Before starting fieldwork, lead researchers should identify project-specific safety concerns, ideally by visiting the study area to develop a field safety plan (see Ramírez-Castañeda and colleagues [1]) that is included in the project handbook (see Rule 6). If a visit is not possible, lead researchers may be able to confer with others who have experience in the area or have conducted similar research to gain insights about potential field safety hazards. Hazards in the field abound and may include weather or topographic concerns such as flash floods, avalanches, or wildfires; biological threats such as large predators, venomous animals, or zoonotic diseases; or navigation challenges in remote areas with few landmarks. For transparency, it is important to disclose any potential concerns to prospective hires during the interview process and discuss how these safety concerns will be mitigated before heading to the field. Field crews should have proper tools and training to collect high-quality data in a safe manner, especially when specialized equipment (e.g., snowmobiles) or methods (e.g., tree climbing) are required for data collection. Lead researchers should keep a record of training procedures that are undertaken, have all crew members sign and certify that they have attended training sessions, and, if possible, have certified instructors provide training. First aid kits that are matched to the local environmental conditions should be provided, and each crew member should be familiar with their contents.

A daily check-in system with someone who is not in the field [7,20] is a critical aspect of field safety. At a minimum, field crews should report the locations in which they are working each day and their expected return time to their check-in contact; once home safely, they should relay that information to their contact who should confirm receipt. There should also be an established, written protocol regarding the steps to take when a crew member fails to check in at their expected time. This written protocol should include contact information for local resources and authorities, including search and rescue teams, local law enforcement, and the location of the closest medical facility. Individuals vary with how well they process information during emergencies so access to written directions can make it easier to follow emergency procedures. If specialized equipment is necessary for check-ins (e.g., satellite safety device), training on such equipment should occur prior to the start of fieldwork. Importantly, all crew members should have multiple methods for navigating in the field (e.g., topographic map, compass, GPS unit) and be able to confidently use those methods under any circumstances.

Lead researchers will benefit by taking an expansive view of safety, including looking beyond the obvious threats or concerns related to working in the field and identifying potential social issues or atypical hazards within the area where field work will take place. For example, they should consider whether a field site would allow someone from a marginalized community to feel safe while working [21] and determine what steps can be taken to mitigate such concerns, such as ensuring that crew members work in pairs. Establishing a regular process of quick and easy check-ins about crew member safety can build trust and promote psychological well-being. If crew members are willing, gather relevant information about their allergies, other medical conditions, or personal risks due to race, religion, gender identity, disability, or sexual orientation, and then work with them to outline specific steps to take if an emergency occurs [22]. If possible, ensure that crew members receive anti-harassment training and, ideally, anti-bias training before fieldwork begins to minimize the occurrence of such incidents and provide clear guidance for appropriate communication, channels for reporting, and crisis support. It is crucial for lead researchers to lead by example and practice the skills learned through those trainings in the field, which helps to emphasize the importance of following all safety protocols.

Finally, it is important to note that some crew members may become complacent about safety once they become comfortable with their work environment, or when working conditions are stressful. Thus, regular reminders about safe working practices throughout the field season are valuable. Starting each work day with a review of potential safety hazards that crew members might encounter keeps safety at the forefront of everyone’s mind. Lead researchers should review any incidents that occur, including during exit interviews, to incorporate lessons learned for the future. Prioritizing and following through with safety measures requires a lot of time and effort, but putting in that work will help lead to a smoother, safer, and more successful field season for the entire crew.

## Rule 6: Create a comprehensive project handbook

When working on a project for the first time, crew members often face a steep learning curve, and we have found that developing a project handbook facilitates onboarding, clarifies expectations, and helps promote consistent data collection throughout a field season. In particular, our experience indicates that crew handbooks are most valuable when they are comprehensive and include (1) an overview of the research being undertaken; (2) the expectations for working safely and safety protocols (see Rule 5); (3) detailed research protocols (see Rule 3); (4) a checklist of required and recommended field equipment; (5) general daily and seasonal work schedules; (6) a section of frequently asked questions and their answers; (7) information about the safety check-in protocol being used (see Rule 5); and (8) a directory of project contacts, including emergency services. Lead researchers should send an electronic version of the handbook to crew members before the field season so they can familiarize themselves with the project’s goals, data collection protocols, and required gear. Once crew members arrive on site, they should receive a hard copy of the handbook to study before going into the field, and a hard copy should be kept in each field vehicle for reference to ensure that a handbook is available without having to rely on an electronic copy. Note that research protocols may need to be adjusted during the field season, particularly in the first year, and it is the responsibility of the lead researcher to update project handbooks to ensure that current protocols are followed and data collection is consistent throughout the project [23].

## Rule 7: Establish a daily routine

Field projects can be complex and stressful, but we find establishing daily routines are very helpful. Scientists have long known the critical role that SOPs play in increasing efficiency, reducing the frequency of errors and accidents, and ensuring consistent, reproducible, and complete data collection [24,25]. Daily routines established before and after fieldwork can play a similar role to SOPs by reducing the stress of leading a complex field project. A routine undertaken before going into the field can ensure that lead researchers and their field crews are physically and mentally prepared for the day ahead. In our experience, such a routine is often centered on a daily checklist used to verify that all materials needed for the day are assembled in place within field packs or vehicles. The checklist should include equipment and consumable materials needed for data collection; a hard copy of the crew handbook (see Rule 6); maps, permits, and keys needed to access study areas; safety and first aid supplies (see Rule 5); and sufficient food and water. If motorized vehicles are used to access field sites, integrate daily vehicle safety assessments (e.g., checking lights, tire pressure) before heading to the field. Finally, it may be beneficial to integrate a short daily meeting prior to the start of each shift, including a review of the day’s itinerary, potential hazards, and strategies to minimize risk exposure (see Rule 5) [26]. The structure of the daily routine will look different for every project and may change over the course of a field season depending on the tasks that are being completed at that time. However, daily communication between crew members and the lead researcher should remain consistent.

After returning from the field, routines are valuable to ensure that data integrity, communication, and organization are maintained. Components of this routine can include crew debriefing, data quality control (see Rule 8), and preparations for the next day. Debriefing can address questions, concerns, and challenges during the day’s fieldwork. A checklist can be helpful for keeping track of daily tasks to complete after a days’ work, such as charging or replacing batteries in battery-operated equipment, printing physical datasheets, cleaning and/or repairing gear, reviewing travel itineraries, and prepping gear, food, and water for the next workday. Making preparations the evening before heading to the field can avoid wasting valuable time in the morning, especially for projects that require leaving particularly early or operate under tight time constraints.

Lead researchers have the responsibility of defining roles for various parts of the daily routine. They should determine who is responsible for daily, weekly, and monthly work scheduling. Daily schedules may be determined by individual crew members, whereas weekly schedules may be determined by crew leaders, and monthly work goals may be set by the lead researcher. Weather conditions can be variable and change quickly so lead researchers should define who makes decisions related to canceling or stopping field work due to weather conditions. Lead researchers may be in charge of assessing weather conditions at the start of the day, whereas individual crew members are responsible for assessing weather conditions throughout the day. Lead researchers should define how communication occurs, whether that is in person or through texts, phone calls, or email. Defining roles and responsibilities for daily routines for all crew members ensures that the field season will operate efficiently in an environment where conditions have the potential to change quickly.

## Rule 8: Engage in good data management practices from the start

Although the process of data collection is important, data management is also essential to ensure that those data will be available in a format that facilitates analysis. Like many of our rules, this one should be implemented well before data collection by establishing data management protocols, including determining which data will be collected and how they will ultimately be used in analyses. Lead researchers should create datasheets and databases in a manner that reduces time spent on data wrangling [27], for example, by structuring databases in a manner that generates “tidy” data as described by Wickham [28].

There are many approaches available for recording data, and lead researchers should assess the pros and cons of multiple approaches before selecting one that best suits their needs. Electronic data collection can reduce data entry time, be automatically backed up, and prevent errors during data entry by requiring data entry forms. However, field-hardy tablets are expensive, may need to be charged daily, and can develop issues related to software. Furthermore, not all digital data are suited for long-term archiving so file types must be considered carefully to ensure that data do not become inaccessible over time if specialized or proprietary programs become unavailable. Under certain field conditions, paper or weatherized materials for data collection may be more durable and reliable, especially if there is inclement weather or extended periods without power. However, physical data sheets may be more challenging to use in windy or rainy weather and their data must be entered manually. Regardless of how data are input, lead researchers should ensure crew members double-check their work to avoid data entry mistakes. This step can be facilitated when using electronic data collection by using forms that only allow preselected values to be placed into databases to eliminate recording and transcription errors. There should also be back-up systems in place for digital and non-digital data (e.g., copies of digital data on hard drives and in cloud storage; photos or scans of field notebooks at the end of each day) and a protocol for controlling and saving different electronic versions of the data [27]. Setting up and following through with organized data management will better prepare the data for subsequent analyses and reduce the likelihood of data loss and the associated stress that comes along with it.

## Rule 9: Be flexible and open to unexpected change

When planning and executing a field season, lead researchers must be flexible and adaptable to change, or at least willing to embrace it. We can attest that fieldwork never proceeds exactly as expected, particularly in the first year of data collection on a new project. For example, data collection may take longer to implement than anticipated, protocols may need to be revised, equipment and vehicles may break down, and crew members may take more time than expected to become efficient with field protocols. These sorts of project “hiccups” should be anticipated as much as possible by lead researchers, but with a recognition that many of the issues that arise in the field are unforeseeable and are beyond the control of anyone on the research team. Investing time before fieldwork to develop sound field protocols often makes it less likely that major protocol changes will be needed during the field season [29]. Considering the likelihood of unknown challenges, it can be helpful to set a minimum level of data to be collected to meet project objectives with the goal of expanding to additional project components as time allows. Importantly, lead researchers should be prepared to drop components that are not critical for project success if they prove to be too time-consuming or logistically infeasible. Of course, modifications to research protocols should be made in consultation with senior project personnel, and these personnel should also be involved in determining which data collection components are immutable and which have flexibility in their timing or extent. If lead researchers do have to alter or adapt data collection during fieldwork, they should keep in mind that there may be benefits to such shifts, including the potential for gaining insights about core components of the study system [2] that may not have been uncovered otherwise. Projects that do not go as planned may also provide unexpected opportunities to write additional papers or research notes, design an alternative pathway to answering a question of interest [2], or even inspire new directions for scientific inquiry.

## Rule 10: Take care of yourself

Fieldwork can be the most enjoyable part of undertaking research but also one of the most stressful and exhausting. Fieldwork is often physically demanding and may require long workdays under a wide range of challenging environmental conditions. Thus, it is essential to make sure that lead researchers are physically and mentally prepared for fieldwork before the start of the season, that they get adequate sleep, nutritious food, and are well-hydrated during the season. Whereas the physical aspect of fieldwork is obvious, those new to leading a field project may not appreciate the mental and emotional challenges that arise from leading a field project. Several factors may impact mental health, including isolation, task loading, deviation from a routine, limited personal space, separation from support networks, and adapting to a new work culture [30]. Lead researchers should recognize that although they are ultimately responsible for a field project, they do not have to take on every task themselves. Instead, they can delegate work to crew members to provide a more even distribution of project responsibilities, particularly for routine tasks. For example, one field crew member might oversee routine maintenance of field vehicles, whereas a second undertaking quality control of data sheets that have been entered into a database. These duties can be rotated throughout the season so that all crew members contribute to the various tasks needed for project success. Sharing responsibility with the crew not only eases the workload for the lead researcher but also provides an opportunity for gaining experience in delegation, which is critical for a career in the sciences.

An important step for lead researchers who rely on mental health services is to create a plan to access the services they need during the field season. Such services may seem inaccessible when working in remote field locations for extended periods, but planning may allow researchers to connect with local care providers or online counseling options. Another way lead researchers can engage in self-care is by intentionally creating separate spaces that support both group interaction and time alone [30]; this separation is particularly important if lead researchers share housing with a field crew.

Finally, maintaining a healthy work–life balance can take a concerted effort when simultaneously being responsible for managing field crews, communicating with collaborators, dealing with unexpected problems, collecting the best data possible, and addressing the many time-sensitive tasks that occur during the field season. Although it can seem counterintuitive, scheduling small periods of time off from fieldwork can be especially valuable for field seasons that may run for several months or more. An increased workload during the field season may seem sustainable because time goes fast during the season and task lists may seem endless. In reality, however, field seasons are often long and draining, and taking care of oneself is crucial during the field season if lead researchers are to maintain physical, mental, and emotional health. Although it is beyond the scope of this set of rules, it is worth mentioning that taking breaks should extend to research that takes place outside of the field, as poor work–life balance is associated with poor mental health conditions among undergraduate students [31], graduate students [32], and professional academics alike [33].

## Concluding thoughts

Fieldwork is often exciting and rewarding for lead researchers but, as we have made clear, implementing a successful field season is a complex and multi-faceted undertaking. It requires substantial forethought, planning, and adequate training for lead researchers and their field crews. Our collective experience has taught us that fieldwork is rife with potential pitfalls, but the rules we have outlined here have helped us successfully navigate many field seasons over the years, and we continue to share these rules with our colleagues who are leading a field crew for the first time. We have found that lead researchers maximize their chances for success when they start the planning process early, are deliberate in assembling and training their crew, clearly communicate their expectations, are flexible in light of adversity, and prioritize their health and self-care. Although fieldwork is often a challenging endeavor—even for those who have extensive experience with it—these rules can make the process a little less daunting for those who are leading a field season for the first time.
